# Expression of integrin α3β1 and cyclooxygenase-2 (COX2) are positively correlated in human breast cancer

**DOI:** 10.1186/1471-2407-14-459

**Published:** 2014-06-20

**Authors:** Anshu Aggarwal, Rami N Al-Rohil, Anupam Batra, Paul J Feustel, David M Jones, C Michael DiPersio

**Affiliations:** 1 Center for Cell Biology & Cancer Research, Albany Medical College, Mail Code 165, Room MS-420, 47 New Scotland Avenue, Albany, NY 12208-3479, USA; 2 Department of Pathology, Albany Medical Center, Albany, NY 12208, USA; 3 Department of Internal Medicine, Albany Medical Center, Albany, NY 12208, USA; 4 Center for Neuropharmacology and Neurosciences, Albany Medical College, Albany, NY 12208, USA

**Keywords:** Integrin α3β1, COX2, PTGS2, Breast cancer, Invasive ductal carcinoma

## Abstract

**Background:**

Expression of integrin α3β1 is associated with tumor progression, metastasis, and poor prognosis in several cancers, including breast cancer. Moreover, preclinical studies have revealed important pro-tumorigenic and pro-metastatic functions for this integrin, including tumor growth, survival, invasion, and paracrine induction of angiogenesis. Our previously published work in a preclinical breast cancer model showed that integrin α3β1 promotes expression of cyclooxygenase-2 (COX2/PTGS2), a known driver of breast cancer progression. However, the clinical significance of this regulation was unknown. The objective of the current study was to assess the clinical relevance of the relationship between integrin α3β1 and COX2 by testing for their correlated expression among various forms of human breast cancer.

**Methods:**

Immunohistochemistry was performed to assess co-expression of α3 and COX2 in specimens of human invasive ductal carcinoma (IDC), either on a commercial tissue microarray (n = 59 samples) or obtained from Albany Medical Center archives (n = 68 samples). Immunostaining intensity for the integrin α3 subunit or COX2 was scored, and Spearman’s rank correlation coefficient analysis was performed to assess their co-expression across and within different tumor subtypes or clinicopathologic criteria.

**Results:**

Although expression of integrin α3 or COX2 varied among clinical IDC samples, a statistically significant, positive correlation was detected between α3 and COX2 in both tissue microarrays (r_s_ = 0.49, p < 0.001, n = 59) and archived samples (r_s_ = 0.59, p < 0.0001, n = 68). In both sample sets, this correlation was independent of hormone receptor status, histological grade, or disease stage.

**Conclusions:**

COX2 and α3 are correlated in IDC independently of hormone receptor status or other clinicopathologic features, supporting the hypothesis that integrin α3β1 is a determinant of COX2 expression in human breast cancer. These results support the clinical relevance of α3β1-dependent COX2 gene expression that we reported previously in breast cancer cells. The findings also suggest that COX2-positive breast carcinomas of various subtypes might be vulnerable to therapeutic strategies that target α3β1, and that α3 expression might serve as an independent prognostic biomarker.

## Background

The most significant cause of mortality in women with breast cancer is metastasis of the primary tumor, and the identification of therapeutic targets to effectively inhibit malignant progression and metastatic spread remains a barrier to the treatment of breast cancer in the clinic. Integrins are the major cell surface receptors for adhesion to the extracellular matrix (ECM), and they are appealing targets for anti-cancer therapies. Indeed, integrins function as bidirectional signaling receptors that regulate both cellular responses to cues from the tissue microenvironment and cell-mediated changes to the microenvironment, and integrin signaling in tumor cells is known to be critically important for promoting malignant growth and metastasis [1-5]. In addition, as cell surface receptors integrins are relatively accessible to inhibitory agents, and several peptide antagonists and humanized monoclonal antibodies that target integrins are in clinical development [2].

All members of the integrin family are transmembrane glycoproteins consisting of an α and a β subunit, where 18 α subunits and 8 β subunits can heterodimerize in different combinations to form 24 distinct integrins with different ligand-binding specificities [3]. The laminin-binding integrin α3β1 is widely expressed in epithelial tissues, including the mammary epithelium, the epidermis, and the kidney glomeruli, where it is important for normal tissue development or function [6-9]. In the normal mammary gland, α3β1 is expressed in both epithelial cells and endothelial cells. Although α3β1 is not required for gross development and differentiation of the mammary gland, genetic deletion of α3 from myoepithelial cells in the lactating mammary gland leads to contractile defects that reduce milk secretion [9,10]. A number of studies have shown that α3β1 promotes tumor growth, invasion, and/or metastasis of breast cancer or other carcinoma cells [11-15]. In addition, two major ECM ligands for α3β1, laminin-332 and laminin-511, are often over-expressed in breast and other carcinomas, and both of these laminins have been linked to tumor invasion and metastasis [16-20]. Indeed, one group’s recent analysis of the Breast Invasive Carcinoma TCGA database revealed a link between decreased patient survival and co-upregulation of the genes encoding the integrin α3 subunit (*ITGA3*) and the laminin α5 chain (*LAMA5*) [15].

Previous studies from our group and others using the triple-negative, aggressive human breast cancer cell line, MDA-MB-231, have shown that integrin α3β1 promotes invasion in vitro and tumor growth in vivo [11,12]. In addition, shRNA-mediated suppression of α3β1 in these cells caused reduced expression of several pro-tumorigenic/pro-invasive genes, including cyclooxygenase-2 (COX2/*PTGS2*) [11]. Furthermore, COX2 was required for some α3β1-mediated cell functions that likely contribute to malignant tumor growth, including invasive potential and pro-angiogenic crosstalk to endothelial cells [11]. These findings have potential clinical significance, as COX2 is a known mediator of breast cancer progression and metastasis that has been an important clinical target of inhibitory therapies [21-23]. Indeed, both non-steroidal anti-inflammatory drugs (NSAIDs) and agents that selectively target COX2 (i.e., celecoxib, rofecoxib, valdecoxib) have been developed [24-26]. However, some COX2 inhibitors produce serious side effects such as gastrointestinal, cardiovascular, liver and kidney complications [27-29], resulting in their voluntary withdrawal from the market in some cases [30,31]. Therefore, exploiting α3β1 as a therapeutic target to down-regulate COX2 gene expression might circumvent certain side effects that have been associated with direct inhibitors of COX2. However, a potential link between α3β1 and COX2 in clinical samples of human breast cancer has not been investigated.

In the current study, we used an immunohistological approach to compare expression of α3 integrin (*ITGA3*) and COX2 (*PTGS2*) among clinical samples of human invasive ductal carcinoma (IDC), and to determine whether there is a correlative relationship between them. Our findings revealed that while the expression of α3β1 varies among clinical samples of IDC, α3β1 showed a statistically significant, positive correlation with COX2 expression. This correlation was detected among tumors of different hormone receptor status, suggesting that α3 expression might serve as an independent prognostic indicator. Together with our earlier findings that α3β1 promotes COX2 expression in breast cancer cells [11], our current data suggest that α3β1 expression may be a determinant of COX2 expression in human breast cancer, and that COX2-positive carcinomas of various subtypes might be vulnerable to therapeutic strategies that target α3β1.

## Methods

### Histological tissue samples

Commercially purchased tissue microarrays (TMAs) included 59 samples of invasive ductal carcinoma (IDC) (Pantomics, Inc., San Francisco, CA, USA; catalog number BRC711), and 12 samples that included normal breast, hyperplasia, IDC, apocrine carcinoma and invasive lobular carcinoma (US Biomax, Inc., Rockville, MD, USA; catalog number T087). In addition, a total of 68 formalin-fixed, paraffin embedded samples of IDC were obtained as archival biopsy material without patient identifiers from the Department of Pathology at Albany Medical Center. Accompanying pathology reports for the latter samples provided information regarding survival status, diagnosis, grade, stage, metastasis of carcinoma, lymph node status and hormone receptor status of patients. This study was approved by the Institutional Review Board of Albany Medical Center.

### Immunohistochemistry

Immunohistology was performed as previously described [32]. Briefly, formalin-fixed paraffin embedded tissues were baked at 55°C for 30 min, then deparaffinized in xylene for 10 minutes and hydrated in an ethanol gradient (100%, 95%, 80%, 70%, distilled water). Tissues were steamed for 30 min in antigen-retrieval solution (Biogenex Laboratories, Fremont, CA, USA), then cooled and washed with 0.1% PBS-BSA solution. Tissue sections were then treated with 3% hydrogen peroxide for 20 minutes, followed by blocking in normal horse serum (Vectastain Elite Kit, Vector Laboratories, Burlingame, CA, USA) for 30 min at room temperature. Tissues were then incubated with rabbit pre-immune serum, or with rabbit polyclonal antiserum against the integrin α3 subunit [33] (1:500 dilution, 1 hr), COX2 (1:200 dilution, 1 hr; Cell Signaling, Danvers, MA, USA) or von-Willebrand Factor (vWF, 1:400 dilution, 30 min; DAKO, Carpenteria, CA) at room temperature, followed by incubation with secondary antibody (Vectastain Elite Kit) for 30 min, then avidin-biotin complex (ABC) for 30 min, according to the manufacturer’s instructions. Specificity of the anti-α3 serum has been demonstrated in previous studies [34,35], and was confirmed under specific conditions of tissue fixation and antigen-retrieval used in the current study by immunostaining of paraffin-embedded sections prepared from neonatal skin of wildtype or α3-knockout mice (data not shown). Sections were stained with 3,3′-diaminobenzidine (DAB; #550880; BD Biosciences, Franklin Lakes, NJ, USA), counterstained with hematoxylin for 20 sec, dehydrated in an ethanol gradient (70%, 80%, 95%, 100%), then immersed in xylene. Sections were mounted using Permount (Sigma, St. Louis, MO, USA) and photographed at 100× magnification using a Nanozoomer (Hamamatsu, Bridgewater, NJ, USA).

### Statistical analysis

Immunohistological staining of breast tissue microarrays for α3 or COX2 was scored blindly by a pathologist using the following criteria: 0 = background, 1 = weakly positive, 2 = moderately positive, 3 = strongly positive. Scores for α3 and COX2 were tabulated, and chi-square tests for trend analyses were performed to analyze the relationship between α3 expression and pathologic diagnostic criteria. Spearman’s rank correlation coefficient analyses were performed to test for a statistically significant positive or negative correlation between α3β1 and COX2 expression across breast cancer subtypes or diagnostic criteria using GraphPad Prism (GraphPad Software, Inc., La Jolla, CA, USA). A p-value of <0.05 was considered statistically significant.

To assess blood vessel density, tumor sections were stained with anti-vWF. Within the region of interest (ROI), the area that stained positive for vWF above a threshold that was set using background staining levels, as determined using IPLab (Scanalytics, Inc., Milwaukee, WI), was averaged between two fields as we described previously [35]. Blood vessel area in relation to the α3 score was analyzed by one-way ANOVA using GraphPad Prism.

## Results

### Analysis of integrin α3β1 expression in clinical breast tumor tissues

**Figure 1:**
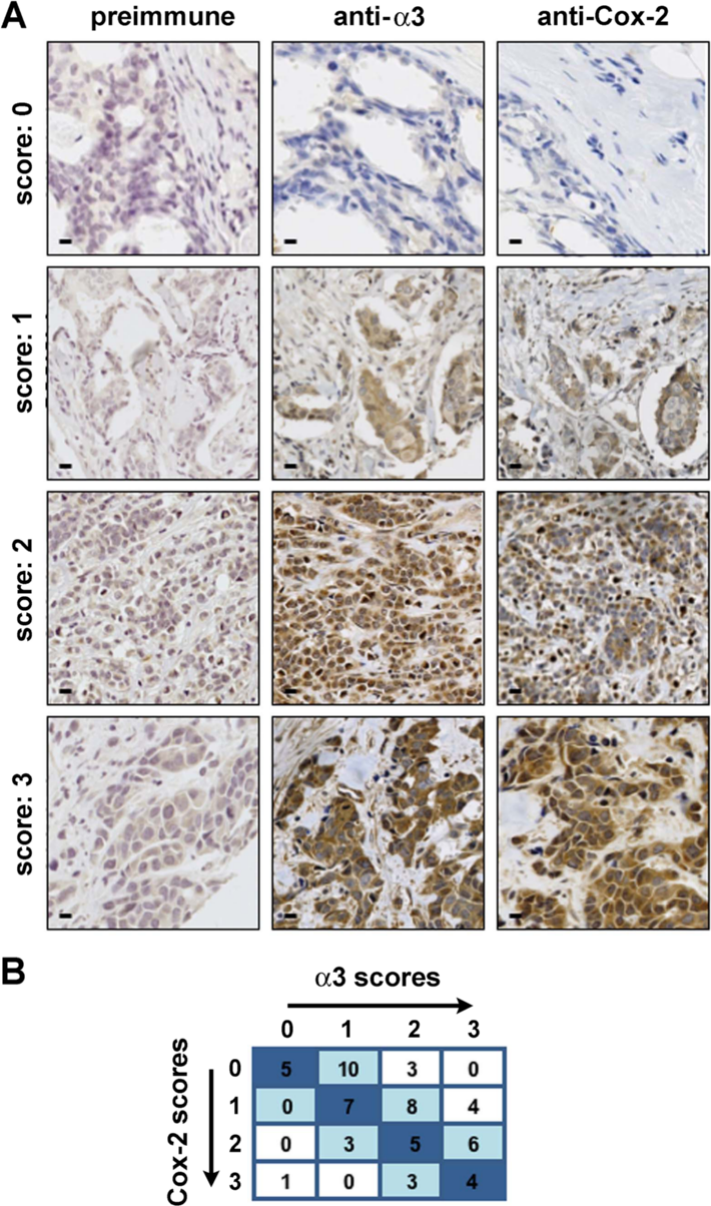
**Expression of integrin α3 and COX2 in human IDC (Pantomics TMA). (A)** Images show representative scoring intensities following immunostaining of adjacent regions from the same tumor with the indicated antibodies (range of 0-3; see Methods). Tissues were also stained with DAB as chromogen, and counter-stained with hematoxylin. The pre-immune serum (first column) was used to determine background staining for each set. Scale bar, 25 μM. **(B)** Table depicts co-distribution of like scores for α3 and COX2. Blue shading highlights a positive correlation for expression of α3 and COX2 among the 59 IDC samples. Spearman’s rank correlation coefficient (r_s_ = 0.49; p < 0.001) indicates a significant correlation between α3 and COX2 expression (see Table 2).

**Figure 2:**
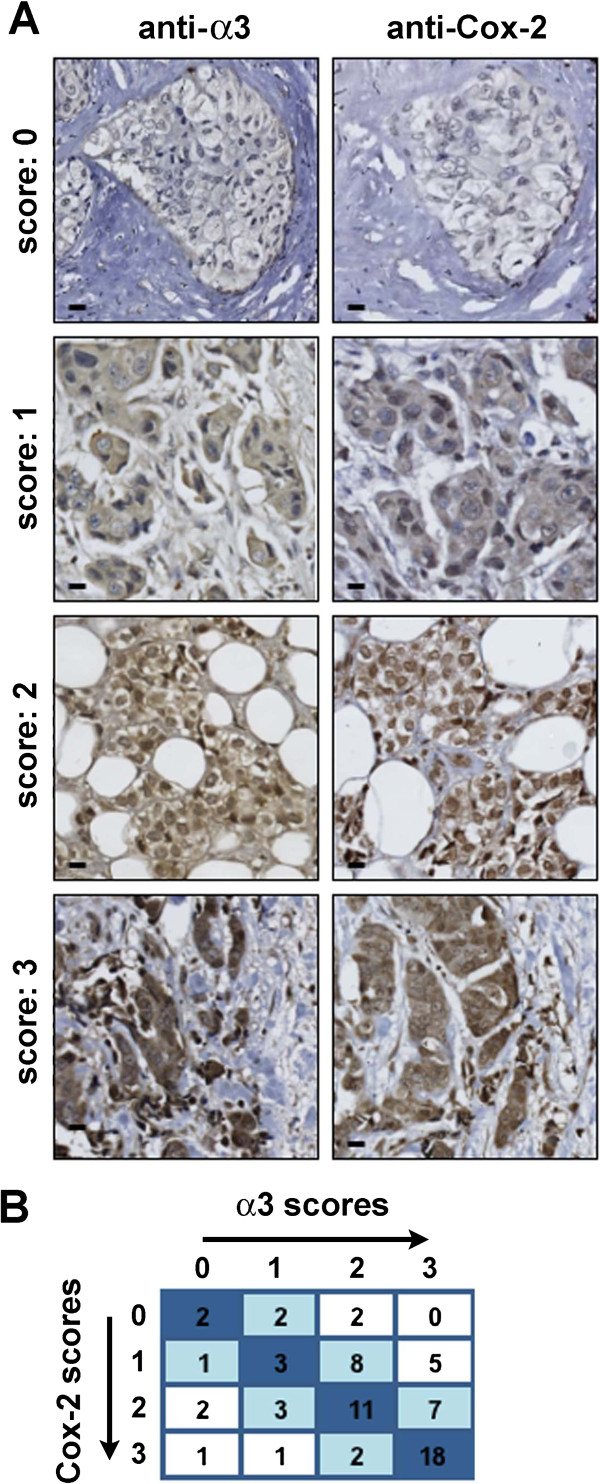
**Expression of integrin α3 and COX2 in human IDC (AMC sample set). (A)** Immunostaining was performed and analyzed as in Figure 1. Images show representative scoring intensities (range of 0-3) for anti-α3 or anti-COX2 of adjacent regions from the same tumor, as indicated. Scale bar, 25 μM. **(B)** Table depicts co-distribution of like scores for α3 and COX2 among the 68 IDC samples, as in Figure 1. Spearman’s rank correlation coefficient (r_s_ = 0.59 and p < 0.0001) indicates a significant correlation between α3 and COX2 expression (see Table 2).

To assess α3β1 expression among breast cancer samples, we first performed immunohistochemistry on commercially available tissue microarrays (TMAs) using an antiserum specific for the integrin α3 subunit (*ITGA3*), or the corresponding preimmune serum from the same rabbit as a calibration control [33]. Importantly, positive staining for the α3 subunit is directly reflective of integrin α3β1 expression, as β1 is the only integrin β subunit with which the α3 subunit dimerizes [3]. Although α3β1 is a cell surface protein, tumors that express this integrin at high levels often show cytoplasmic staining of the α3 subunit, presumably reflecting α3 that has not reached the cell surface or has been internalized [36,37]. Consistently, α3 staining was observed in the cytoplasm of the tumor cells, as well as in some of the surrounding endothelial cells.After anti-α3 immunostaining was calibrated against background staining obtained with the preimmune serum (Figure 1A, preimmune column), all samples were blindly scored for α3 staining intensity on a scale of 0 (no staining) to 3 (high staining) (Figure 1A, anti-α3 column; see Methods for details). Analysis of a TMA from Pantomics revealed variable α3β1 expression among 59 independent cases of IDC, of which 6 (10%) showed no staining, 20 (34%) showed low staining, 19 (32%) showed medium staining, and 14 (24%) showed high staining. Examples of variable α3 expression are shown in Figure 1A (compare preimmune and anti-α3 columns). Immunostaining of a smaller TMA containing 12 tissues (US Biomax), including normal mammary tissue, ductal hyperplasia, invasive lobular carcinoma (ILC), apocrine carcinoma, and IDC revealed similarly variable α3 staining (data not shown).We next expanded our analysis to 68 IDC samples obtained from the tissue bank at the Albany Medical Center (AMC) Pathology Department, which included data regarding tumor grade, lymph node status, metastasis, and survival status. Analysis of these AMC samples revealed similarly variable α3β1 expression, where 6 (9%) showed no staining, 9 (13%) showed low staining, 23 (34%) showed medium staining, and 30 (44%) showed high staining. Among these tissues, α3 staining was again detected at varying degrees of intensity in the cytoplasm of tumor cells, as well as in some stromal cells (Figure 2A, anti-α3 column).

As shown in Table 1, a statistically significant association of α3 staining in the Pantomics array was observed with tumor grade (p = 0.027; chi-square test for trend) and HER2 (human epidermal growth factor receptor 2) status (p = 0.013), but not with ER (estrogen receptor) status, PR (progesterone receptor) status, or tumor stage. However, similar analysis of the AMC sample set did not reveal a statistically significant association of α3 staining with tumor grade or HER2 status, nor with stage, although in these samples we did detect an association with ER (p = 0.015) and PR status (p = 0.036). We did not detect an association of α3 expression with tumor metastasis, tumor recurrence, or nodal status. We also did not observe a statistically significant trend of α3 staining with triple-negative (i.e., HER2-/ER-/PR-) status (data not shown), although this sample set was limited to only 13 samples. Thus, while trends were observed within certain clinicopathologic groupings, these trends did not consistently reach statistical significance in both the Pantomics TMA and the AMC sample sets.

**Table 1:** Contingency tables for α3 or COX2 scores versus clinicopathology

			**α3 scores**				**Cox-2 scores**			
	**0**	**1**	**2**	**3**	**p-value**	**0**	**1**	**2**	**3**	**p-value**
**Pantomics TMA**										
**Histological grade**										
I (n = 2)	1	0	0	1	0.027*	1	0	1	0	0.64
II (n = 22)	5	6	5	6		6	9	3	4	
III (n = 35)	0	14	14	7		11	10	10	4	
**Stage**										
Early (n = 47)	4	16	14	13	0.25	13	15	11	8	0.16
Advanced (n = 12)	2	4	5	1		5	4	3	0	
**HER2**										
Negative (n = 21)	4	9	6	2	0.013*	10	7	3	1	0.014*
Positive (n = 38)	2	11	13	12		8	12	11	7	
**ER**										
Negative (n = 33)	3	13	12	5	0.28	12	11	7	3	0.14
Positive (n = 26)	3	7	7	9		6	8	7	5	
**PR**										
Negative (n = 29)	3	11	10	5	0.39	11	10	6	2	0.08
Positive (n = 30)	3	9	9	9		7	9	8	6	
**AMC samples**										
**Histological grade**										
I (n = 10)	0	1	2	7	0.34	1	2	3	4	0.39
II (n = 35)	2	4	13	16		2	8	10	15	
III (n = 23)	4	4	8	7		3	7	10	3	
**Stage**										
Early (n = 44)	2	5	15	22	0.06	5	9	15	15	0.89
Advanced (n = 24)	4	4	8	8		1	8	8	7	
**HER2**										
Negative (n = 50)	5	7	16	22	0.64	6	12	15	17	0.59
Positive (n = 18)	1	2	7	8		0	5	8	5	
**ER**										
Negative (n = 23)	3	5	10	5	0.015*	5	7	10	2	0.001*
Positive (n = 45)	3	4	13	25		1	11	13	20	
**PR**										
Negative (n = 32)	4	6	12	10	0.036*	4	11	10	7	0.027*
Positive (n = 36)	2	3	11	20		2	6	13	15	
**Metastasis**										
No (n = 64)	5	8	22	29	0.73	5	17	22	20	0.82
Distant (n = 4)	1	0	1	2		1	0	1	2	
**Nodal status**										
0 (n = 32)	2	4	10	16	0.34	4	7	12	9	0.49
1 (n = 36)	4	5	13	14		2	10	11	13	
**Tumor recurrence**										
No (n = 49)	4	7	14	24	0.48	4	9	18	18	0.08
Yes (n = 19)	2	2	9	6		2	8	5	4	

The distribution of α3 or COX2 staining intensity scores (range 0-3, see Methods for details) is shown for various clinicopathologic features. Chi-square tests for trend were performed on the Pantomics TMA to test for a significant relationship between α3 expression and tumor grade, disease stage, or hormone-receptor status. AMC samples were additionally assessed with regard to metastasis, lymph node status, and tumor recurrence. The same tests were performed for COX2 expression. *p < 0.05 is considered statistically significant; all significant values are shown in bold.

### Analysis of COX2 expression in clinical breast tumor tissues

Staining with an antiserum specific for COX2 (*PTGS2*) was also variable among IDC samples, as shown in Table 1, and illustrated in Figures 1A and 2A (anti-COX2 columns). Indeed, analysis of the Pantomics TMA revealed that among the 59 IDC samples, 18 (30%) showed no staining, 19 (32%) showed low staining, 14 (24%) showed medium staining, and 8 (14%) showed high staining. Similarly, COX2 staining was variable among the 68 AMC samples, with 6 (9%) showing no staining, 17 (25%) showing low staining, 23 (34%) showing medium staining, and 22 (32%) showing high staining.

As shown in Table 1, a statistically significant association of COX2 expression was seen with HER2 status (p = 0.014; chi-square test for trend), but not with tumor grade, ER status, PR status, or stage. However, similar analysis of the AMC samples did not reveal a statistically significant association of COX2 staining with HER2, but did detect an association with ER (p = 0.001) or PR status (p = 0.027). Interestingly, despite the differences between the Pantomics TMA and AMC sample set, the significant trends observed for COX2 expression within each sample set were also seen for α3 staining (Table 1), suggesting that α3 staining and COX2 staining might be correlated (see below). We did not detect a statistically significant association of COX2 staining with tumor grade, stage, tumor metastasis, nodal status, or tumor recurrence.

### Expression of COX2 is correlated with expression of α3β1 in human breast cancer

Our previous study showed that integrin α3β1 expression in MDA-MB-231 human breast cancer cells promotes invasion and tumor growth in part through the induction of COX2 gene expression [11]. Therefore, we next wanted to determine whether α3β1 expression is positively correlated with COX2 expression in human breast cancer samples. For these analyses, sections from adjacent regions of the same tissue were scored for cytoplasmic staining intensity of either α3 or COX2, using the 0 to 3 scale described above. Spearman’s rank correlation coefficient analyses were then performed to compare staining intensity between sections and test for a statistically significant correlation between α3β1 and COX2 expression patterns.

Initial analysis of TMAs (Pantomics or US Biomax) showed similar staining of α3 and COX2 in epithelial cells of both normal breast tissue and breast tumor tissue, as well as in some of the surrounding stromal cells (data not shown). Analysis of the Pantomics TMA revealed a statistically significant correlation between α3β1 and COX2 expression among IDC samples (Table 2; Spearman’s rank correlation coefficient r_s_ = 0.49, p < 0.001, n = 59). Representative images in Figure 1A illustrate the similar staining patterns and intensities for α3 and COX2 in adjacent regions of the same tumors (compare paired panels in anti-α3 and anti-COX2 columns). Data regarding the histological grade, tumor stage and hormone receptor-status were provided by Pantomics, which had been scored previously by the manufacturers on a similar 0 to 3 scale. Spearman’s rank correlation coefficient analysis of each group (i.e., HER2-negative vs. HER2-positive) revealed that a statistically significant, positive correlation between α3β1 and COX2 expression was observed irrespective of the hormone receptor status, histological grade, or stage of the cancer (Table 2).

**Table 2:** Correlation of COX2 and α3 among IDC samples of different subtype or clinicopathology

	**r**_**s**_	**p-value**
**Pantomics TMA**	**0.49**	< 0.001*
All samples (n = 59)		
**Histological grade**		
I (n = 2)	N/D	N/D
II (n = 22)	**0.61**	0.002*
III (n = 35)	**0.55**	0.001*
**Stage**		
Early n = 47	**0.56**	< 0.001*
Advanced n = 12	**0.72**	0.008*
**HER2**		
Negative n = 21	**0.82**	< 0.001*
Positive n = 38	**0.47**	0.003*
**ER**		
Negative n = 33	**0.6**	< 0.001*
Positive n = 26	**0.57**	0.003*
**PR**		
Negative n = 29	**0.67**	< 0.001*
Positive n = 30	**0.49**	0.006*
**AMC samples**		
All samples (n = 68)	**0.59**	< 0.0001*
**Histological grade**		
I (n = 10)	**0.69**	0.039*
II (n = 35)	**0.53**	0.001*
III (n = 23)	0.27	0.22
**Stage**		
Early n = 44	**0.54**	< 0.001*
Advanced n = 24	0.49	0.05
**HER2**		
Negative n = 50	**0.54**	< 0.001*
Positive n = 18	0.4	0.1862
**ER**		
Negative n = 23	**0.63**	0.001*
Positive n = 45	**0.34**	0.02*
**PR**		
Negative n = 32	**0.53**	0.002*
Positive n = 36	**0.42**	0.01*
**Metastasis**		
No (n = 64)	**0.49**	< 0.001*
Distant (n = 4)	0.63	N/D
**Nodal status**		
0 (n = 32)	**0.49**	0.005*
1 (n = 36)	**0.51**	0.002*
**Tumor recurrence**		
No (n = 49)	**0.49**	< 0.001*
Yes (n = 19)	0.3	0.21

Spearman’s rank correlation coefficient (r_s_) tests were performed on the Pantomics TMA to test for correlation between α3 and COX2 expression within all samples (n = 59), or within subgroups of various clinicopathologic features including tumor grade, disease stage, or hormone receptor status. AMC samples (n = 68) were also assessed together, or within the same subgroups, as well as with regard to metastasis, lymph node status, and tumor recurrence. N/D, no data. *p < 0.05 is considered statistically significant; all significant values are shown in bold.

We performed similar analysis of the AMC IDC samples. However, these tissue sections were considerably larger than the focal regions provided on the commercial TMAs. Therefore, we first selected an area of tumor cells within each tissue section that showed the most intense cytoplasmic staining with the COX2 antiserum as the “region-of-interest” (ROI), which was assigned a score of 0 (background staining) to 3 (intense staining). Staining intensity for α3 was then scored similarly on a 0 to 3 scale in the corresponding ROI of an adjacent region from the same tissue. As was seen for the Pantomics TMA, analysis of the AMC sample set revealed a statistically significant correlation between α3β1 and COX2 expression (Table 2; Spearman’s rank correlation coefficient r_s_ = 0.59, p < 0.0001, n = 68). Figure 2A shows representative images illustrating this correlation (compare paired panels), which was observed regardless of ER, PR or nodal status (Table 2). In addition, this correlation was statistically significant for HER2-negative, lower grade, and early stage samples, and it approached significance in advanced stage tumors (p = 0.05). Taken together, our results indicate that expression of COX2 is positively correlated with expression of α3β1 in clinical samples of human IDC. Moreover, this relationship holds regardless of hormone receptor status, and within tumors of different histological grade or stage.

**Figure 3:**
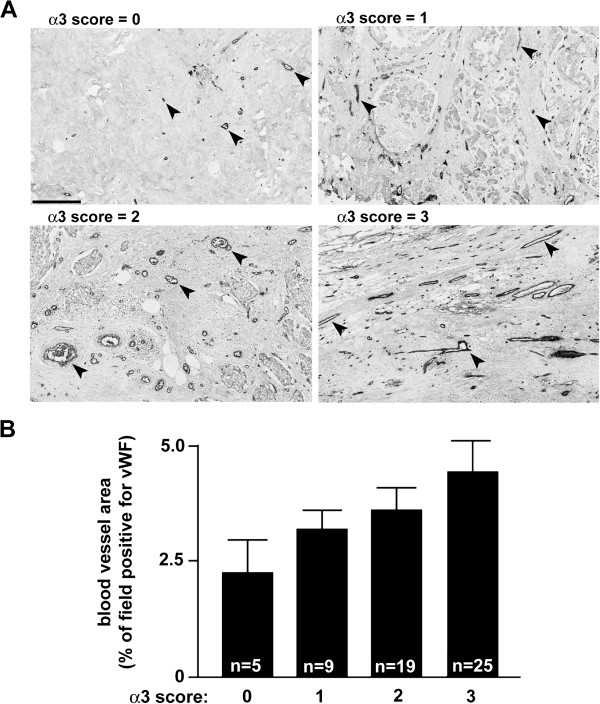
**Assessment of blood vessel density in human IDC with varying α3 expression (AMC sample set). (A)** Representative examples of anti-vWF immunostaining among tumor samples of varying α3 score, as indicated. Arrowheads point to examples of blood vessels. Scale bar, 250 μM. **(B)** Graph depicts quantification of blood vessel area (i.e., anti-vWF staining above threshold) among tumor samples of varying α3 expression score, as indicated. Data are average +/- s.e.m.; sample size is indicated for each bar on the graph.

Since we recently reported that expression of α3β1 in breast cancer cells is correlated with enhanced tumor angiogenesis in a preclinical xenograft model [11], we also assessed the AMC IDC samples for a relationship between α3 expression and blood vessel density. Blood vessel area within tumor sections was determined by quantification of anti-vWF immunostaining using IPLab (Scanalytics, Inc.), as we have described [35], then compared across sample groups with α3 staining scores ranging from 0 to 3 (one-way ANOVA). Although differences in blood vessel density among the groups were not statistically significant, interestingly we did observe an overall trend of elevated blood vessel density with increased expression of α3 (Figure 3).

## Discussion

The current study corroborates earlier findings that integrin α3β1 is detected in a large proportion of human breast cancers [12], although its expression levels vary considerably [38]. Importantly, our findings also identify a novel, positive correlation between the expression of α3β1 (assessed by staining for the α3 subunit) and COX2 in clinical samples of human IDC, thereby validating the clinical relevance of our earlier report that α3β1 regulates COX2 expression in a preclinical breast cancer model [11]. The potential translational impact of these findings lies in the fact that COX2 is already a well known promoter of breast cancer progression and tumor angiogenesis that has been exploited in the clinic as a therapeutic target [21-23,39]. Importantly, however, COX2 inhibitors often produce severe side effects that include gastrointestinal complications and increased cardiovascular risks [24,27-29]. Our current findings support the inhibition of integrin α3β1 as a promising therapeutic strategy, as this approach may provide an alternative mode of suppressing COX2 without the adverse side effects that have been associated with direct COX2 inhibitors.

The general concept of targeting integrins to inhibit cancer progression is already established, and clinical testing of peptide antagonists (e.g., the RGD mimetic cilengitide) and humanized monoclonal antibodies that target certain integrins is well underway [2,40]. However, most of these agents are currently directed against integrins that are expressed on endothelial cells and promote tumor angiogenesis, such as αvβ3 and αvβ5 [2,41]. In contrast, strategies to inhibit the functions of tumor cell integrins are relatively underdeveloped, in part due to a critical need to identify and validate the most appropriate integrins to target on particular types of cancer cells. A formidable barrier towards this goal is that the repertoire of integrins expressed by tumor cells varies across different types of cancer, and different integrin αβ heterodimers have distinct functions [3]. Indeed, clinical studies have revealed different expression patterns for individual β1 integrins in breast cancer, where expression of some integrins (e.g., α3β1) increases or persists compared with normal tissue [12], while expression of other integrins (e.g., α2β1) often decreases [42]. Furthermore, a recent study in a preclinical model identified α2β1 as a suppressor of breast cancer metastasis [42], in contrast with the pro-tumorigenic functions that have been described for other β1 integrins such as α3β1 and α6β1 [11,43,44], emphasizing the need to identify individual integrins with cancer-promoting roles that would be appropriate to exploit as therapeutic targets.

Importantly, the results of our current study, combined with our previous preclinical study [11], provide support for α3β1 as a promising therapeutic target on breast cancer cells. Indeed, the role that α3β1 plays in promoting COX2 gene expression extends to other genes with pro-tumorigenic/pro-metastatic roles [11,45], including MMP-9 [12,46], suggesting that blocking the gene regulatory functions of this integrin might suppress multiple tumor cell functions that drive carcinogenesis. α3β1 has been implicated as a potential marker protein for cells undergoing enhanced EMT or for cancer cells with aggressive phenotypes [37], and the transcription factor Ets-1 may play role in transcriptional activation of the α3 subunit gene [47]. Moreover, studies performed in both genetic models and xenograft models have revealed important roles for α3β1 in promoting tumorigenic or metastatic behavior of breast cancer cells. For example, α3β1 has been shown to promote malignant growth of basal mammary epithelial cells through activation of intracellular signaling pathways that involve FAK, Rac1/PAK1, MAPK and JNK [48]. In addition, orthotopic implantation of aggressive breast cancer cell lines in which α3β1 was suppressed using RNAi displayed significant reductions in primary tumor growth [11,15], as well as a dramatic reduction of spontaneous or experimental metastasis [15], indicating important and potentially separable roles for this integrin at both early stages of tumorigenesis and later stages of metastasis.

Despite the above progress in preclinical models, little is known about the importance of α3β1 within different breast cancer subtypes, or whether α3β1 expression is correlated to clinical diagnostic characteristics such as hormone receptor status, tumor stage, or metastasis. Although our analysis detected trends of increased α3 expression in IDC of certain hormone receptor status, these trends did not reach statistical significance in both the AMC samples and the commercial TMA, so their significance remains uncertain. We obtained similarly variable results in our analysis of COX2 expression across IDC samples of distinct hormonal status, consistent with varying reports of the relationship between COX2 and hormone receptor status or other diagnostic criteria. For example, in one report COX2 activation was associated with ER-negative and HER2-positive breast cancers, while in another it was positively associated with ER and PR status [49,50]. We also failed to detect significant associations of either α3 or COX2 expression with tumor stage, tumor grade, recurrence, nodal status, or metastasis. Importantly, however, the positive correlation that we detected between α3 expression and COX2 expression was statistically significant within subgroups of distinct hormone receptor status, histological grade, or tumor stage, indicating that this correlation is not associated with any particular IDC subtype or stage. These findings suggest that targeting α3β1 to inhibit COX2 expression might be an effective therapeutic strategy for various forms of IDC that express COX2.

While the potential for α3β1 as a useful therapeutic target for breast cancer is clear, it is important to note that some studies have indicated suppressive roles for α3β1 in certain cancer models, indicating that pro-tumorigenic functions of this integrin may be context-dependent [51-53]. Indeed, while shRNA-mediated silencing of α3β1 in breast cancer cells reduced cell invasion in vitro and tumor growth in vivo [11,15], similar silencing of α3β1 enhanced lung metastasis in an in vivo model of prostate cancer [53]. Moreover, α3β1 expression varied considerably among breast tumors, as shown here and by others [12,38]. Interestingly, results from in vitro and in vivo models have indicated that some α3β1 functions are acquired by some immortalized/transformed cells [46] or may be associated with distinct stages of progression within a cancer type [54], indicating that functions of this integrin may change during cancer development and progression. For example, a recent study in a squamous cell carcinoma model showed that epidermis-specific deletion of α3β1 (i.e., using a conditional α3-knockout model) led to reduced skin tumorigenesis, whereas tumors that did form in these mice progressed more readily to invasive carcinoma, indicating opposing roles for α3β1 in early and late stages of skin carcinogenesis [54].

## Conclusions

In summary, our finding that expression of integrin α3β1 and COX2 are correlated in human IDC is likely to reflect an important physiological role for the α3β1-dependent regulation of COX2 gene expression that we described previously in cultured breast cancer cells [11,45]. Together, these findings support the concept that targeting α3β1 specifically on tumor cells may provide an alternative strategy of suppressing COX2 that circumvents adverse side effects associated with current COX2 inhibitors. This approach might be broadly applicable to different breast cancer subtypes, since the correlation between α3 expression and COX2 expression was not associated with any particular hormone receptor status. Another potential benefit of this approach stems from the ability of α3β1 to regulate other pro-tumorigenic/pro-metastatic genes [11,45], which suggests that inhibiting this integrin on tumor cells might produce the effect of a multi-target approach.

## Abbreviations

COX2: Cyclooxygenase-2; ECM: Extracellular matrix; IDC: Invasive ductal carcinoma; TMA: Tissue microarray; AMC: Albany Medical Center; HER2: Human epidermal growth factor receptor 2; ER: Estrogen receptor; PR: Progesterone receptor.

## Competing interests

The authors declare that they have no competing interests.

## Authors’ contributions

All authors participated in the design of the project. AA performed all immunohistology of tissue sections, image acquisition, and statistical analysis, and drafted the manuscript. PJF assisted with study design and statistical analysis. RNA-R and DMJ evaluated tumor sections and scored immunostaining intensities. AB assisted with immunohistochemical analysis of blood vessel density. CMD conceived of the study, coordinated the project, and was involved in writing the manuscript. All authors read and approved the final manuscript.

## Pre-publication history

The pre-publication history for this paper can be accessed here:

http://www.biomedcentral.com/1471-2407/14/459/prepub
